# Psychosocial factors of caregiver burden in child caregivers: results from the new national study of caregiving

**DOI:** 10.1186/s12955-015-0317-2

**Published:** 2015-08-07

**Authors:** Steven A. Cohen, Sarah Cook, Lauren Kelley, Trisha Sando, Allison E. Bell

**Affiliations:** Division of Epidemiology, Department of Family Medicine and Population Health, Virginia Commonwealth University School of Medicine, 830 E. Main Street, 8th Floor, P.O. Box 980212, Richmond, VA 23298-0212 USA; Department of Internal Medicine, Virginia Commonwealth University School of Medicine, Richmond, VA USA

## Abstract

**Background:**

Over 50 million informal caregivers in the United States provide care to an aging adult, saving the economy hundreds of billions of dollars annually from costly hospitalization or institutionalization. Despite the benefits associated with caregiving, caregiver stress can lead to negative physical and mental health consequences, or “caregiver burden”. Given these potential negative consequences of caregiver burden, it is important not only to understand the multidimensional components of burden but to also understand the experience from the perspective of the caregiver themselves. Therefore, the objectives of our study are to use exploratory factor analysis to obtain a set of latent factors among a subset of caregiver burden questions identified in previous studies and assess their reliability.

**Methods:**

All data was obtained from the 2011 National Study of Caregiving (NSOC). Exploratory factor analysis (EFA) was performed to identify a set of latent factors assessing four domains of caregiver burden in “child caregivers”: those informal caregivers who provide care to a parent or stepparent. Sensitivity analysis was also conducted by repeating the EFA on demographic subsets of caregivers.

**Results:**

After multiple factor analyses, four consistent caregiver burden factors emerged from the 23 questions analyzed: Negative emotional, positive emotional, social, and financial. Reliability of each factor varied, and was strongest for the positive emotional domain for caregiver burden. These domains were generally consistent across demographic subsets of informal caregivers.

**Conclusion:**

These results provide researchers a more comprehensive understanding of caregiver burden to target interventions to protect caregiver health and maintain this vital component of the US health care system.

## Introduction

Over 50 million American adults, age 18 and over, provide informal care to an adult over the age of 50 [1]. Americans spend over 30 billion hours per year providing informal care to disabled or chronically ill individuals with an opportunity cost of $522 billion per year [2]. This figure is only expected to rise as the percent of the population 65 years and older grows from 40 million to 55 million by the year 2020 [3]. Additionally, the population of those over the age of 85 is expected to grow to over 6.5 million by 2020 and to over 19 million by 2050, thus increasing the proportion of those who need access to more intensive and costly care [3].

Adult children of care recipients, or “child caregivers”, make up nearly half of individuals providing informal care [4], of which a majority struggle to divide their time and resources among their own immediate family, work and providing direct and indirect care for their loved one. Maintaining these multiple responsibilities over time can result in caregiver stress. Although the care provided by informal caregivers benefits both the care recipient and society, the effects of caregiver stress can lead to negative physical and mental health consequences [5–9]. In a national survey of caregivers, 17 % reported “fair” or “poor” health compared to only 13 % of the general population [1]. Additionally, 31 % reported that caregiving was an emotionally stressful experience and 7 % responded as “dissatisfied” or “very dissatisfied with life” [10].

Caregiving can also have a negative social and financial impact on the caregiver. Fifty-three percent of caregivers reported that caregiving duties took away from their family and friends, 89.2 % had less leisure time, and 23 % reported that caregiving resulted in financial hardship [1, 11, 12]. Caregiver stress in child caregivers can also have indirect effects on employment [13, 14], childcare [15, 16], and marital relationships [17]. This combination of health, emotional, financial, and social consequences of caregiving are commonly referred to as “caregiver burden.” Caregiver burden is defined as a negative reaction to the impact of providing care on caregivers’ social, occupational, and personal roles [18] and can lead to burnout and other negative consequences, potentially impacting their ability to provide care.

With the potential for negative consequences, it is important to better understand how informal child caregivers are affected by the caregiving experience. Understanding the multidimensional components of caregiver burden can guide research and, ultimately, interventions designed to improve and maintain the quality of life among this critical component of the US health care system [19]. Additionally, it is important to better understand the child caregiver experience from the perspective of the caregiver rather than the one receiving care, in order to craft policies and programs aimed at improving the caregiver experience.

Recently the National Heath and Aging Trends study published the data from the initial National Study of Caregiving (NSOC). This is the first survey to examine the entire caregiver experience in the US population. Currently, the NSOC evaluates the following components of a caregiver’s experience: care activities, duration of care, aspects of caregiving, support environment, participation, health, employment, and caregiving. The NSOC also collects caregiver demographic characteristics such as household composition, health insurance status, and income. To date, no analysis has been conducted to determine the latent structure of caregiver burden domains using a nationally representative survey of child caregivers. Therefore, there were two objectives of our analysis. First, we sought to identify latent constructs of the potentially multidimensional aspects of caregiver burden among child caregivers. Second, we assessed the consistency of those latent factors among subpopulations of child caregivers.

## Methods

### Data source

The data were obtained from the 2011 National Study of Caregiving (NSOC) dataset, which contains information on approximately 2000 individuals responsible for providing some form of care to an elderly family member or friend. Participants in the National Health and Aging Trends Study (NHATS) identified up to five individuals as caregivers. These caregivers provide help with household activities such as preparing meals, shopping, or transportation. They also assist the NHATS participant with personal care such as help with medications, bathing, or dressing [20]. The identified caregivers were contacted to participate in a one-time, cross-sectional assessment of caregiving. Our primary analysis was restricted to adult children caregivers (*n* = 1014) in the NSOC dataset.

### Measures

Demographic characteristics of the respondents such as age, gender, marital status, education level, and poverty status were preliminarily assessed. Additionally, the caregiver’s relationship with the care recipient and whether the caregiver had a child under 18 in the home were described.

To measure caregiving burden, items from the sections of the questionnaire pertaining to aspects of caregiving including health, health insurance and income, and social participation were used. It also included questions pertaining to emotional well-being such as feelings of cheerfulness, boredom, loneliness, depression, anxiety, and peacefulness. The section pertaining to health insurance and income was used to determine the financial burden of caregiving, particularly as it pertained to the caregiver’s use of personal funds. Personal funds provided general financial assistance to the care recipient or to assist in paying for medical expenses such as medications, health insurance, in-home help, and mobility devices. Social participation was assessed using the respondent’s reported participation in activities including: religious services, going out with friends, visiting with family and friends, and volunteering.

### Statistical analysis

Descriptive analyses were performed on the demographic variables to characterize the study population. In addition to the descriptive analysis, exploratory factor analysis (EFA) was performed to identify a set of latent factors assessing caregiver burden from the larger set of caregiver burden questions identified previously. EFA was performed using the method of principal components analysis using orthogonal varimax rotation selection on 31 variables. Scree plots, eigenvalues (>1.0), differences in model variance, and loading scores (>0.4) were used to decide the number of factors. Additional analysis was conducted using a subset of those 31. A sensitivity analysis was then conducted, repeating the EFA using the entire sample of caregivers, on demographic subsets of caregivers: males, females, adult children of care recipients with children under age 18 in the home (“sandwiched” caregivers), and without children under age 18 in the home (non-“sandwiched” caregivers).

## Results

### Sample characteristics

The primary analytical sample consisted entirely of sons and daughters caring for an aging parent (*n* = 1,014, 69 % of which were female and 31 % were male. Of the study participants, 54 % were married, 17 % were divorced, and 19 % were never married. Additionally, almost 18 % of the sample had children under the age of 18 and 24 % had no living children. The mean age of the sample was approximately 55 years of age with a minimum age of 19 and a maximum age of 77. The majority (58 %) had at least some college education and another 25 % had a high school diploma. Just under one-fifth of the caregivers (19.5 %) reported “excellent” health, while 18.2 % reported “fair” or “poor” health.

### Exploratory factor analysis

During the initial analyses on all 31 variables, 8 variables did not load distinctly on any factor and had eigenvalues less than 1. These variables were removed from the analysis. Analysis of the remaining items indicated a seven factor solution with 52.1 % of the variance explained; however, the Cronbach’s alpha for the final factor was low (0.38). Analyses of these same items were then conducted with a six then five factor solution. The percent variance explained decreased to 47.5 % for a six factor solution to 42.5 % with a five factor solution, and the Cronbach’s alpha remained to appear not significant. A four factor solution was then analyzed; however, many of the items had loading scores less than 0.4 and the percent variance explained dropped to 37.4 %. Items with loadings less than 0.4 were removed from the analysis along with one item with a significant amount of missing responses. After removing these items, analysis of a four factor solution of the remaining items was repeated and selected as the final model.

The first factor (eigenvalue = 5.45 and variability = 28.71 %) had high loadings for variables associated with negative emotions such as being nervous, worried, feeling down, feeling upset, and having little interest in things, and was named “negative emotional” (Table 1). The second factor (eigenvalue = 2.68 and variability = 14.13 %) exhibited high loadings for items associated with positive emotions such as feeling confident, cheery, full of life, and peaceful. This factor was labeled “positive emotional”. The third factor (eigenvalue = 1.38 and variability = 7.27 %) exhibited high loadings for variables associated with social participation such as participating in group activities, going out, attending religious services, volunteering, and visiting family and friends. As a result, this factor was labeled “social”. Lastly, the fourth and final factor (eigenvalue = 1.09 and variability = 5.79 %) had high loadings for financial aspects of caregiving such as providing financial support and money for medications and other medical devices, and was labeled “financial”. With the inclusion of these four factors, approximately 46 % of the variance was explained.

**Table 1:** Results from best exploratory factor analysis model on adult children caregivers

	Factor number				
	1	2	3	4	Name of factor
Down & depressed	0.756				*Negative emotional*
Nervous	0.745				
Worried	0.642				
Little interest	0.574				
Upset	0.522				
Lonely	0.504				
Peaceful		0.819			*Positive emotional*
Full of life		0.803			
Cheerful		0.766			
Group activities			0.713		*Social*
Volunteers			0.601		
Religious services			0.536		
Visiting family & friends			0.496		
Out for enjoyment			0.483		
Paid for meds/med care				0.759	*Financial*
Gave CR gift				0.748	
*Cronbach’s a*	*0.710*	*0.790*	*0.510*	*0.461*	

### Sensitivity analysis

We then assessed the consistency of these caregiver burden domains across subsets of informal caregivers: all caregivers, females, males, “sandwiched” adult children caregivers, and non-“sandwiched” adult children caregivers using the items selected in the final four factor solution Results were generally consistent across caregiver subsets. Questions associated within each domain remained identical in each subset, (all items loading scores for each subset >0.4) (Table 2). Reliability of latent factors ranged from 0.368 in males (financial burden) to 0.737 in non-“sandwiched” child caregivers (negative emotional burden). The reliability of the 4th factor (financial) decreased in male (0.368), females (0.455), and non-sandwiched adult children caregivers (0.384) subgroups, and increased in sandwiched adult children caregivers (0.503). The percent variance explained by the EFA model was fairly constant across caregiver subgroups, ranging from 46.5 % in males to 48.4 % in “sandwiched” caregivers.

**Table 2:** Sensitivity analysis of caregiver burden domains by caregiver subgroup

	All caregivers	Males	Females	Sandwiched adult children caregivers	Non-sandwiched adult children caregivers	
Down & depressed	0.756	0.771	0.755	0.745	0.762	1. Negative emotional
Nervous	0.745	0.725	0.748	0.710	0.753	
Worried	0.642	0.575	0.671	0.667	0.638	
Little interest	0.574	0.553	0.588	0.541	0.570	
Upset	0.522	0.543	0.523	0.425	0.560	
Lonely	0.504	0.457	0.513	0.388	0.542	
Peaceful	0.819	0.810	0.819	0.819	0.816	2. Positive emotional
Full of life	0.803	0.810	0.795	0.818	0.806	
Cheerful	0.766	0.807	0.738	0.780	0.780	
Group activities	0.713	0.679	0.726	0.673	0.708	3. Social
Volunteers	0.601	0.667	0.601	0.475	0.592	
Religious services	0.536	0.635	0.501	0.571	0.509	
Visiting family & friends	0.496	0.446	0.487	0.542	0.508	
Out for enjoyment	0.483	0.416	0.460	0.455	0.477	
Paid for meds/med care	0.759	0.672	0.771	0.731	0.769	4. Financial
Gave CR gift	0.748	0.669	0.663	0.683	0.752	
Worked for pay	0.504	0.539	0.502	0.612	0.429	
Cronbach’s alpha	0.710	0.661	0.730	0.696	0.737	Factor 1
	0.790	0.797	0.786	0.809	0.792	Factor 2
	0.510	0.502	0.511	0.496	0.496	Factor 3
	0.461	0.368	0.455	0.503	0.384	Factor 4
% variance explained	46.4	46.5	46.8	48.4	46.6	

## Conclusion

The domains of child caregiver burden that emerged from the factor analysis (positive emotional, negative emotional, social, and financial) are consistent with findings from previous studies on caregivers which assessed the different and often coinciding positive and negative effects of caregiving [12, 19, 21, 22]. The social burden domain exhibited with the factor analysis points to the isolation and disruption that may occur when serving as a caregiver. Other studies have shown that caregivers who are able to participate socially in activities outside of caregiving are better able to cope with the stresses of caregiving compared to those who give up their social activities [12]. Participation in social activities may potentially alleviate the reported social burden of caregiving. It is also possible that engaging in social activities may lessen the negative emotional impact of caregiving.

The financial burden domain is again in line with previous findings. In 2009, 27 % of adult caregivers reported that they experienced a moderate to high financial burden as a result of caregiving [1]. Surveys have shown out-of-pocket costs related to caregiving averaged $5,500 annually for all informal caregivers [23]. With respect to the negative emotional domain, caregiving and the magnitude of burden can have negative emotional effects on caregivers, perhaps through the process of causing life disruption as a result of the caregiving process [1, 11, 12, 19].

However, the effects of caregiving are not entirely negative. Consistent with prior studies, our results suggest that caregiving can have a positive influence on the caregiver [22]. Caregivers may receive personal satisfaction by feeling valued, learning new skills, and building relationships with family members and friends [19].

There are important limitations to consider in this analysis. First, this study sample of caregivers focused primarily on adult children of care recipients. The experience of adult children may be inherently different from those who care for a friend, grandparent, spouse, or another relative. However, the majority of informal caregivers are adult children [4]. We also conducted a sensitivity analysis using other sample subsets. In that analysis, the burden domains were fairly consistent across these demographic groups. A second limitation is that this analysis did not account for caregiving intensity. Higher caregiving intensity is associated with increased caregiver burden [24]. It is possible that caregivers providing higher levels of care may experience caregiver burden differently than caregivers who provide lower levels or lower frequency of care.

Despite these limitations, this study highlights the patterns of burdens that child caregivers face, and provide important areas to consider for the health and well-being of an important sub-group of health care providers. Developers of health care policy and programs should consider exploring and targeting the identified domains (negative emotional, positive emotional, social, and financial) to ensure new policy has a positive impact on informal child caregivers and provides a more supportive environment for these individuals. As the population continues to age, older adults will more heavily contribute to the overall rise in health care costs in the United States. Medicare costs alone are projected to rise to 20 % of the GDP in 2050 [25]. However, informal caregivers, particularly child caregivers, play a critical role in decreasing this costly utilization. They not only provide direct care, which decreases overall health care utilization, but caregivers are often able to maintain community-based living arrangements for care recipients, thereby delaying the care recipient’s transition to more intensive and costly long-term care [26]. Nationally, the savings resulting from informal caregiving totals more than 75 % of annual Medicare spending [11]. Therefore, improved understanding of caregiver burden is essential to ultimately maintain and even strengthen this vital component of the health care system as the population continues to age.

The exploratory analysis presented may help inform future research in this field in several important ways. First, this analysis represents the first such exploratory analysis of caregiver burden domains specifically in child caregivers. As the population continues to age and with increasing intergenerational age gaps, the number of child caregivers to elderly parents will continue to increase. The needs of child caregivers who may simultaneously balance work and child caregiving obligations are distinct from those of spousal and other types of informal caregivers. Second, this preliminary analysis is among the first to explore the new National Study of Caregiving, which offers a unique and comprehensive examination of informal caregivers across the United States. Lastly, this preliminary analysis explored the multidimensionality of caregiver burden in child caregivers to elderly parents. Identifying potential domains of burden can inform future research in informal caregiving and highlights the breadth of consequences, both positive and negative, of informal caregiving above and beyond physical effects.
